# Hot Rocks and Not-So-Hot Rocks on the Seashore: Patterns and Body-Size Dependent Consequences of Microclimatic Variation in Intertidal Zone Boulder Habitat

**DOI:** 10.1093/iob/obz024

**Published:** 2019-10-09

**Authors:** A R Gunderson, M Abegaz, A Y Ceja, E K Lam, B F Souther, K Boyer, E E King, K T You Mak, B Tsukimura, J H Stillman

**Affiliations:** 1 Estuary & Ocean Science Center, Romberg Tiburon Campus, San Francisco State University, 3150 Paradise Drive, Tiburon, CA 94920, USA; 2 Department of Integrative Biology, University of California, 1005 Valley Life Sciences Building #3140, Berkeley, CA 94720-3140, USA; 3 Department of Biology, California State University, Fresno, CA 93740, USA; 4 Department of Biology, San Francisco State University, 1600 Holloway Avenue, San Francisco, CA 94132, USA; 5 Department of Ecology and Evolutionary Biology, Tulane University, New Orleans, LA 70118, USA

## Abstract

Microclimatic variation has emerged as an important driver of many ecological and evolutionary processes. Nonetheless, fine-scale temperature data are still rare in most habitats, limiting our ability to understand the consequences of microclimatic variation under current and future conditions. We measured fine-scale thermal variation in a common, species-rich, but rarely studied habitat with respect to temperature: the airspaces under rocks on intertidal zone boulder shores. The effects of thermal variation were investigated using physiological, behavioral, and demographic responses of the porcelain crab *Petrolisthes cinctipes*. Habitat temperatures were measured at fine spatial and temporal resolution over 18 months, producing 424,426 temperature records. Microclimatic variation increased with increasing intertidal elevation, particularly with respect to heat extremes. However, mean temperatures were similar across the entire intertidal zone. Overheating risk for *P. cinctipes* increases with intertidal elevation but is size dependent, as large animals are more heat sensitive than small animals. Still, microclimatic variation high in the intertidal zone provided thermal refugia even under the warmest conditions. Size-dependent thermal responses predicted that large crabs should be rare high in the intertidal zone, which was supported by demographic data. Furthermore, simulations parameterized by our microclimate and organismal data recapitulated demographic patterns. Therefore, interactions between microclimatic variation and size-dependent thermal responses may have significant ecological repercussions that warrant greater attention.

## Synopsis

Rocas calientes y rocas no tan calientes en la orilla del mar: patrones y consecuencias dependientes del tamaño del cuerpo de la variación microclimática en el hábitat de rocas intermareales (Hot rocks and not-so-hot rocks on the seashore: patterns and body-size dependent consequences of microclimatic variation in intertidal zone boulder habitat) La variación microclimática se ha convertido en un importante impulsor de muchos procesos ecológicos y evolutivos. No obstante, los datos de temperatura a pequeña escala aún son raros en la mayoría de los hábitats, lo que limita nuestra capacidad de comprender las consecuencias de la variación microclimática en las condiciones actuales y futuras. Medimos la variación térmica a pequeña escala en un hábitat común, rico en especies, pero rara vez estudiado con respecto a la temperatura: los espacios aéreos debajo de las rocas en las costas de rocas de zonas intermareales. Los efectos de la variación térmica se investigaron utilizando respuestas fisiológicas, conductuales y demográficas de los cangrejos de porcelana *Petrolisthes cinctipes*. Las temperaturas del hábitat se midieron con una resolución espacial y temporal fina durante 18 meses, produciendo 424.426 registros de temperatura. La variación microclimática aumentó con el aumento de la elevación intermareal, particularmente con respecto a los extremos de calor. Sin embargo, las temperaturas medias fueron similares en toda la zona intermareal. El riesgo de sobrecalentamiento de *P. cinctipes* aumenta con la elevación intermareal, pero depende del tamaño, ya que los animales grandes son más sensibles al calor que los animales pequeños. Aún así, la variación microclimática alta en la zona intermareal proporcionó refugios térmicos incluso en las condiciones más cálidas. Las respuestas térmicas dependientes del tamaño predijeron que los cangrejos grandes deberían ser raramente altos en la zona intermareal, lo que fue respaldado por datos demográficos. Además, las simulaciones parametrizadas por nuestros microclimas y datos organismales recapitularon patrones demográficos. Por lo tanto, las interacciones entre la variación microclimática y las respuestas térmicas dependientes del tamaño pueden tener importantes repercusiones ecológicas que justifican una mayor atención. Translated to Spanish by YE Jimenez (yordano_jimenez@brown.edu)

Rochas quentes e rochas não-tão-quentes à beira-mar: padrões e conseqüências dependentes do tamanho coporal para a variação microclimática no habitat de rochas da zona entremarés (Hot rocks and not-so-hot rocks on the seashore: patterns and body-size dependent consequences of microclimatic variation in intertidal zone boulder habitat) A variação microclimática emergiu como um importante causador de muitos processos ecológicos e evolutivos. No entanto, dados de temperatura em escala fina ainda são raros para a maioria dos habitats, limitando nossa capacidade de compreender as conseqüências da variação microclimática sob condições atuais e futuras. Nós medimos a variação térmica numa escala fina em um habitat comum, rico em espécies, mas raramente estudado em relação à temperatura: os espaços aéreos sob as rochas nas margens dos blocos de pedra da zona entremarés. Os efeitos da variação térmica foram investigados usando respostas fisiológicas, comportamentais e demográficas do caranguejo *Petrolisthes cinctipes*. As temperaturas do habitat foram medidas em resolução espacial e temporal ao longo de 18 meses, produzindo 424.426 registros de temperatura. A variação microclimática aumentou com o aumento da elevação intertidal, particularmente com relação aos extremos de calor. No entanto, as temperaturas médias foram semelhantes em toda a zona entremarés. O risco de superaquecimento de *P. cinctipes* aumenta com a elevação intertidal, mas é dependente do seu tamanho, já que os animais grandes são mais sensíveis ao calor do que os pequenos. Ainda assim, a variação microclimática alta na zona entremarés forneceu refúgios térmicos mesmo sob as condições mais quentes. Respostas térmicas dependentes do tamanho previam que os grandes caranguejos deveriam ser raros na zona entremarés, o que era apoiado por dados demográficos. Além disso, as simulações parametrizadas pelos nossos dados de microclima e organismal recapitularam padrões demográficos. Portanto, as interações entre a variação microclimática e as respostas térmicas dependentes do tamanho corporal podem ter repercussões ecológicas significativas que merecem maior atenção. Translated to Portuguese by G Sobral (gabisobral@gmail.com)

Heiße Felsen und weniger heiße Felsen an der Küste: Muster und körpergrößenabhängige Folgen der mikroklimatischen Variation im Felsenhabitat der Gezeitenzone (Hot rocks and not-so-hot rocks on the seashore: patterns and body-size dependent consequences of microclimatic variation in intertidal zone boulder habitat) Die mikroklimatische Variation ist als wichtiger Treiber vieler ökologischer und evolutionärer Prozesse hervorgetreten. Allerdings sind detaillierte Temperaturdaten für die meisten Habitate noch selten, was unsere Fähigkeit einschränkt, die Konsequenzen mikroklimatischer Schwankungen unter gegenwärtigen und zukünftigen Bedingungen zu verstehen. Wir haben die thermische Variation hochaufgelöst in einem gewöhnlichen, artenreichen, aber in Bezug auf die Temperatur selten untersuchten Habitat gemessen: den Lufträumen unter Felsbrocken in der Gezeitenzone von Felsküsten. Die Auswirkungen der thermischen Variation wurden anhand physiologischer, verhaltensbezogener und demographischer Reaktionen der Porzellankrabbe *Petrolisthes cinctipes* untersucht. Die Habitattemperaturen wurden über 18 Monate mit feiner räumlicher und zeitlicher Auflösung gemessen, wobei 424.426 Temperatureinträge erstellt wurden. Die mikroklimatische Variation nahm mit zunehmender Höhe im Gezeitenbereich zu, insbesondere in Bezug auf Hitzeextreme. Die mittleren Temperaturen waren jedoch über die gesamte Gezeitenzone ähnlich. Das Überhitzungsrisiko für *P. cinctipes* steigt mit zunehmender Höhe im Gezeitenbereich, ist jedoch größenabhängig, da große Tiere hitzeempfindlicher als kleine Tiere sind. Trotzdem bot die mikroklimatische Variation in der oberen Gezeitenzone auch unter den wärmsten Bedingungen einen Temperaturschutz. Größenabhängige thermische Reaktionen sagten voraus, dass große Krebse in der oberen Gezeitenzone selten sein sollten, was durch demographische Daten gestützt wurde. Darüber hinaus haben Simulationen, mit unseren Mikroklima- und Organismendaten als Parametern, demographische Muster wiedergegeben. Daher können Wechselwirkungen zwischen mikroklimatischen Schwankungen und größenabhängigen thermischen Reaktionen erhebliche ökologische Auswirkungen haben, die größere Aufmerksamkeit erfordern. Translated to German by F Klimm (frederike.klimm@biologie.uni-freiburg.de)

## Introduction

Temperature is one of the most important abiotic factors shaping the ecology and evolution of organisms (Angilletta 2009; Somero et al. 2017), and it is changing across the world (IPCC 2014). Although climate change is a global phenomenon, environmental temperature can vary extensively at fine spatial (from millimeters to meters; Huey et al. 2009; Caillon et al. 2014) and temporal (from minutes to days; Wang and Dillon 2014) scales, with important ecological consequences (Kearney et al. 2012; Hannah et al. 2014; Montalto et al. 2014; Colinet et al. 2015; Sears and Angilletta 2015; Woods et al. 2015; Gunderson et al. 2016; Williams et al. 2016). For example, female lizards that live a few meters apart but with different basking opportunities have different seasonal reproductive patterns (Otero et al. 2015), while survival of extreme cold in insects is linked to the frequency and duration of daily cold exposure (Marshall and Sinclair 2015). Effects of fine-scale thermal variation extend to communities, where it facilitates niche partitioning and species coexistence (e.g., Stillman and Somero 2000; Kaspari et al. 2015; Gunderson et al. 2018). Nonetheless, most investigations consider thermal environments at broad spatial and temporal scales relative to the scales at which organisms experience them (Potter et al. 2013). As a result, there is a pressing need to increase our understanding of microclimatic variability and its consequences, particularly in light of ongoing climate change (Suggitt et al. 2018).

Marine intertidal zones are among the most thermally dynamic habitats on Earth due to daily patterns of immersion and emersion. Emersion subjects intertidal organisms to terrestrial sources of thermal variability, including air temperature, wind, and increased radiation (Helmuth 1998; Bonicelli et al. 2014); as a result, intertidal organisms commonly experience rapid temperature change (Harley and Helmuth 2003). The physical structure of intertidal surfaces also creates fine-scale spatial temperature variation. For example, animals on shaded rocky surfaces can be significantly cooler than those on nearby exposed surfaces (Denny et al. 2006; Jackson 2010; Seabra et al. 2011; Miller and Dowd 2017). The thermal variation within the intertidal zone can have major physiological implications: a few centimeters can mean the difference between experiencing benign or lethal temperatures (Helmuth and Hofmann 2001; Harley 2008; Denny et al. 2011; Jimenez et al. 2015; Mislan and Wethey 2015).

Most studies of fine-scale intertidal thermal variation focus on exposed surfaces of rocky bench shorelines and the organisms that specialize on those habitats, such as mussels, limpets, and snails (Gilman et al. 2006; Harley et al. 2009; Miller and Denny 2011; Helmuth et al. 2016; see also citations in previous paragraph). In contrast, relatively little is known about fine-scale thermal variability and its consequences in other intertidal habitats. One such habitat is intertidal boulder fields, characterized by limited sand and covered with medium to large rocks. The shaded, moist air spaces under rocks in these globally prevalent shorelines provide habitat for a diverse array of animals including fish, crustaceans, mollusks, poriferans, bryozoans, cnidarians, echinoderms, and polychaetes (Ríos and Mutschke 1999; Le Hir and Hily 2005; Kuklinski and Barnes 2008; Chapman 2012; Liversage et al. 2017). What are the thermal environments like in these habitats, and how does temperature shape the ecology and evolution of the organisms that live there?

In their now classic “hot rocks and not-so-hot rocks” study, Huey et al. (1989) used retreat-site selection in garter snakes to demonstrate the importance of thermal variation under terrestrial rocks. They found that snakes non-randomly chose retreat rocks with respect to temperature, favoring those with physical features that allow them to stay cool enough to avoid overheating, but warm enough to maintain physiologically beneficial body temperatures. Our goal was to determine if similar processes are at play for the diverse organisms under intertidal zone rocks and, if so, to explore their ecological repercussions. We investigated fine-scale temperature variation in an intertidal under-boulder habitat by measuring temperature under rocks across a range of intertidal elevations over 18 months on a single shore (424,426 temperature records total). The consequences of thermal variation were measured on *Petrolisthes cinctipes*, a small suspension-feeding porcelain crab that occurs under rocks and in mussel beds (Morris et al. 1980). We focused specifically on energetics, overheating risk, behavioral heat avoidance, and demographic structure by integrating field observations, laboratory experiments, and simulations. Our primary findings are that (1) thermal extremes under rocks vary consistently with intertidal elevation and in some cases rock size, (2) despite being protected from direct solar radiation, heat extremes can exceed the thermal limits of organisms that live under rocks, (3) spatial variation in temperature among rocks provides opportunity for behavioral thermoregulation to avoid overheating, (4) spatial patterns of crab body size distribution across the intertidal zone can be explained by interactions between fine-scale thermal variation and body-size dependent physiology and behavior. Our results highlight the importance of accounting for both environmental variation within habitats and phenotypic variation among individuals when trying to understand how populations are shaped by, and will respond to change in, abiotic conditions.

## Methods

### Habitat temperature

Data were collected on a south-facing shore near Fort Ross State Park in northern California (38°30′45.79′′N, 123°14′45.58′′W; see Supplementary Fig. S1) from June 19, 2015, through December 12, 2016, except February 1, 2016 to April 3, 2016, when storms rolled boulders and prevented data collection. For descriptive purposes, we categorized our study area into two zones: mid intertidal zone (MIZ; *N* = 12; intertidal elevation range = −0.32–0.23 m; mean = 0.0 m), and high intertidal zone (HIZ; *N* = 12; elevation range = 0.31–1.10 m; mean = 0.8 m); elevations are relative to mean lower low water. MIZ rocks sustained extensive algal growth on upper surfaces during summer, whereas HIZ rocks had sparse or no algal cover. Intertidal elevations were calculated with ground survey methods that employ yardsticks and a basic theodolite (Mason et al. 2000), with reference points set by observed lower low water level on a calm day based on tide charts. The length, width, and height of each rock were measured, and size ranged from 568 to 2,671 cm^2^ surface area and 5162 cm^3^ to 50,472 cm^3^ volume.

Temperature data loggers (iButton DS1921G, Dallas Semiconductor) in waterproof brass casings (23 mm × 25 mm) were affixed to the center of the underside of rocks with Z-spar marine epoxy, occupying the area where *P. cinctipes* occur (personal observation). Sampling occurred at 0.5°C accuracy every 30 min, except from June 19 to August 1, 2015 (every 15 min). Data were downloaded every 2–4 weeks as tides allowed. Temperatures recorded on deployment and retrieval days were eliminated to avoid effects of handling. Temperatures were also removed if a rock flipped and the iButton was exposed to solar radiation.

### Demographic sampling of *P. cinctipes*

To sample demographics, a rock was flipped over and all crabs were collected and placed in a container with fresh seawater. Carapace width was measured at the widest point between the first two walking legs to assign crabs to one of four size bins (<7 mm, 7.1–10.0 mm, 10.1–13.0 mm, >13.0 mm). The rock was then replaced and the crabs were released under it. We marked and returned to the same rocks for each sampling event, with the exception of January 22 and February 20, 2016, when some unmarked rocks were sampled. Sampling effort changed over the course of the study (Supplementary Table S1). In 2015, 3–5 rocks were sampled in the MIZ on each date, while 9–12 were sampled in the HIZ. In 2016, 11–15 MIZ rocks and 9–20 HIZ rocks were sampled per trip.

### Energetic expenditure

Baseline aerobic metabolism was estimated with published *P. cinctipes* resting oxygen consumption rates at different temperatures (15°C–30°C at 5°C intervals) in air and water (Stillman and Somero 1996; see Supplementary Fig. S4). Data were available for small (0.9 g, ∼7 mm carapace width) and large (4.3 g, ∼17mm carapace width) crabs. We calculated the best-fit line of log mass-specific metabolic rate versus temperature for each body size, with separate models fit for metabolism in air and water. Metabolic rates were not calculated for temperature records above the mean heat tolerance of *P. cinctipes* (30.5°C, see “Results” section) as they would cause an animal to perish or move.

For each field temperature, a baseline metabolic rate was calculated for a large and small animal. Air or water metabolic rates were applied depending on the predicted water height at the time of the temperature record (Helmuth et al. 2002; Miller et al. 2015). Time-dependent tide height data were extracted for Point Arena, CA (approximately 40 miles north of our site) using the *rtides* package in R, and then corrected by known differences in the timing and magnitude of tidal events between Fort Ross and Point Arena (code in Supplementary Appendix S1). Instantaneous metabolic rates and cumulative energetic expenditure were calculated for each rock each day. Cumulative calculations assumed animals experienced a given temperature for the duration of the interval between temperature records (Miller et al. 2015).

### Specimen collection and maintenance

Crabs used for physiological and behavioral assays of heat tolerance, preferred temperature, and heat avoidance were collected from a boulder field at Fort Ross ≥200 m away from our habitat temperature and demographics sampling area. Prior to use in experiments, crabs were maintained for ≥2 weeks in laboratory aquaria held at a temperature of 13°C ± 0.5°C and a salinity of 33 ± 3 ppt and were fed a mix of marine microalgae (Shellfish Diet 1800, Reed Mariculture Inc., Campbell, CA) every 2–3 days.

### Heat tolerance

Heat tolerance (*N* = 40 crabs) was measured as the Arrhenius break point temperature for cardiac performance (Stillman 2003; Stillman 2004). Briefly, two 25 µm copper electrodes were inserted into the dorsal surface of the carapace on either side of the heart. Electrodes were connected to impedance converters (UFI 2991, Morro Bay, CA) connected to a PowerLab16sp data acquisition system (ADinstruments, Colorado Springs, CO) with LabChart software (chart v.5). Data were recorded as voltage and converted to beats per minute. Crabs were placed into circulating aerated and temperature-controlled seawater at 12°C. Following a 30 minute recovery from handling, temperature was increased by 0.1°C/min up to 36°C. Cardiac break point temperature (CT_max_) for each individual was estimated as the temperature at the intersection of best-fit lines fit the first 200 and final 60 (prior to flatline) data points (Stillman 2003; Stillman 2004).

### Preferred temperature

Preferred temperatures (*T*_pref_; *N* = 17) was measured in an aluminum thermal gradient (150 cm × 9 cm × 5 cm) in which water temperature ranged from 6°C to 30°C, creating a gradient of 0.16°C/cm (Supplementary Fig. S2). Each end of the gradient was connected to an aluminum block with embedded copper tubing through which water was pumped from temperature-controlled water baths. The gradient bar was covered with red semi-transparent acrylic (porcelain crabs have poor visual sensitivity to long wavelength light; Ziedens and Meyer- Rochow, 1990) to provide a dark environment that porcelain crabs favor. Crabs were individually placed in the 13°C region of the gradient and allowed 30 min to acclimate before measurements began. Water temperature at the crab’s location was measured with a digital thermometer (Omega model HH603A, type T probe) using a wire temperature probe every 15 min for 3 h, with the mean of these temperatures taken as the crab’s *T*_pref_. Probes were threaded through a small opening running lengthwise down the acrylic cover such that it did not have to be removed, minimizing disturbance to the crabs. Two crabs remained at one end of the gradient during the entire trial, one at the hot end, the other at the cold end, and were removed from the analysis.

### Behavioral heat avoidance

Thermal escape temperature (*T*_esc_) was measured as the temperature at which crabs voluntarily exited a temperature chamber during a thermal ramp (*N* = 17, size range = 10–17 mm) using methods broadly similar to those used for other intertidal crabs (e.g., McGaw 2003). The chamber was a petri dish (10 cm diameter × 1.5 cm height) filled to 1 cm depth with aerated, filtered seawater, and nested in an aluminum block (15 cm × 15 cm; Supplementary Fig. S3). The block was fitted with internal copper tubing (outer 3/8″) connected to a water bath by flexible polyvinyl chloride pipe (inner 3/8″) allowing the temperature to be ramped. A ceramic plate was placed 4 cm above the surface to provide a preferred dark environment relative to the bright area around the plate. Crabs were placed in the dish for 10 min at 13°C before the temperature ramp to acclimate to the chamber. The temperature was then ramped at 0.5°C/min while water temperature was monitored with a digital thermometer. *T*_esc_ was recorded as the water temperature when the crab fully exited the petri dish.

### Simulations of intertidal size structure

To determine if size-dependence of CT_max_ and *T*_esc_ are sufficient to produce a *P. cinctipes* body size distribution similar to what we observed in the field, we conducted individual-based simulations using the modeling software NetLogo (V5.3.1). We simulated a rocky intertidal zone of 2000 rocks, with 100 horizontal columns of “rocks” (i.e., perpendicular to the shore) and 20 vertical rows of rocks (i.e., parallel to the shore). The 10 highest rows were set as the HIZ, while the bottom 10 rows were set as the MIZ. Each day in the simulation, each rock was assigned a temperature drawn at random from the distributions of daily maximum temperatures we observed under rocks from the same intertidal zone. We included temporal structure in simulated temperatures by restricting available temperatures on a given simulated day to field temperatures measured on that day or 1 day before or after (e.g., temperatures on August 25 could only come from maximum rock temperatures measured August 24–26). Temperature data for the same calendar day on different years were combined. Simulations ran for 1 year from January 1 to December 31, minus the days with no temperature data, for 295 days total.

Each simulation was seeded with *N* = 1000 crabs ranging from 4 to 21 mm carapace width, with an equal number of crabs in each of our four demographic size bins. Initial crab positions were random. Each crab was assigned a *T*_esc_ and a CT_max_ based on body size using the best-fit lines from our experimental data (see “Results” section). On a given day if a crab experienced a temperature above *T*_esc_, it moved to a random adjacent rock, and continued moving until it found a temperature below *T*_esc_. A crab might not always move when *T*_esc_ is reached due to factors such as the presence of predators and competitors, leaving the possibility that it could experience lethal temperatures. To account for this, we conducted simulations in which movement probability upon reaching *T*_esc_ was below one (i.e., 0, 0.2, 0.4, 0.6, and 0.8). We also conducted simulations with the same set of probabilities that a crab would perish upon experiencing its CT_max_. These variations in probability of response also allowed us to determine how robust our results are to different strengths of size dependence (i.e., the lower the probability of a size-specific response, the lower the strength of size-dependence). We ran 1000 simulations for each combination of movement and survival probability. Simulation code is available online (http://modelingcommons.org/browse/one_model/5980).

### Statistical analyses

We tested for effects of rock characteristics (intertidal elevation and rock surface area) on under-rock thermal conditions (mean maximum, mean, and mean minimum temperatures) using linear models with the “lm” function in R (v. 3.3.3, R Core Team 2017). We predicted that intertidal elevation would associate positively with under-rock temperatures whereas rock size would be negatively associated with under rock temperatures. Tests of the relationships between body size and thermal tolerance, preferred temperature, and escape temperature were also conducted with linear models.

## Results

### Habitat temperatures

**Fig. 1:**
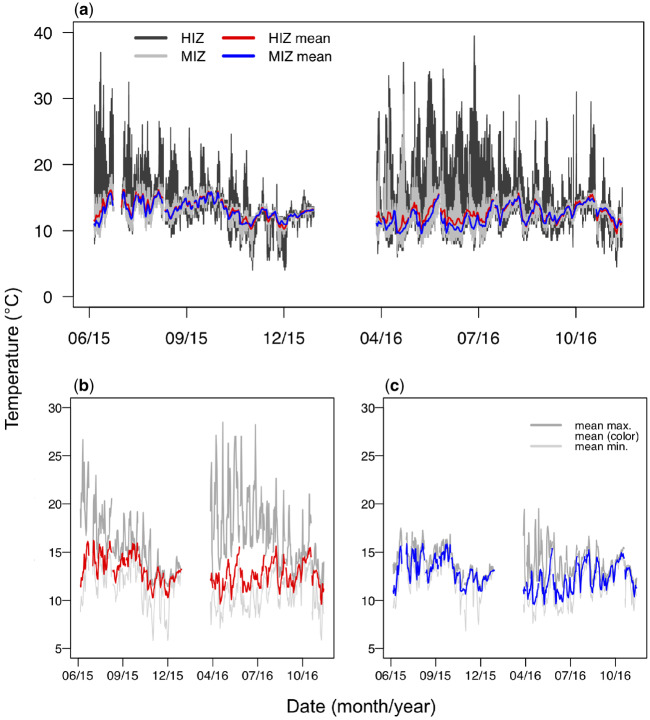
Temperatures recorded under intertidal rocks. (**a**) All temperatures recorded in the HIZ and MIZ along with daily mean temperatures. (**b**) Mean daily maximum and minimum temperatures in the HIZ and (**c**) in the MIZ.

The mean temperature under HIZ rocks was 0.3°C warmer than under MIZ rocks (X± standard deviation [SD]; *X*_MIZ_ = 12.7°C ± 1.7°C, *X*_HIZ_ = 13.0 ± 2.4). Zones differed more in daily maxima, with HIZ rocks (16.4°C ± 4.1°C) 2.5°C warmer than MIZ rocks (13.9°C ± 1.8°C; Fig. 1b and c). In contrast, mean minimum temperatures in the HIZ (11.2°C ± 2.0°C) were 0.5°C cooler than in the MIZ (11.7°C ± 1.8°C; Fig. 1b and c). Thermal maxima were higher in spring/summer than in fall/winter, though the effect was much greater in the HIZ (18.8°C ± 4.4°C versus 14.7°C ± 2.5°C) than in the MIZ (14.2°C ± 2.1°C versus 13.7°C ± 1.3°C) (Fig. 1). HIZ rocks averaged a greater daily temperature range (5.2°C ± 4.5°C) than those in the MIZ (2.2°C ± 1.8°C), particularly in the summer (Fig. 1b).

**Fig. 2:**
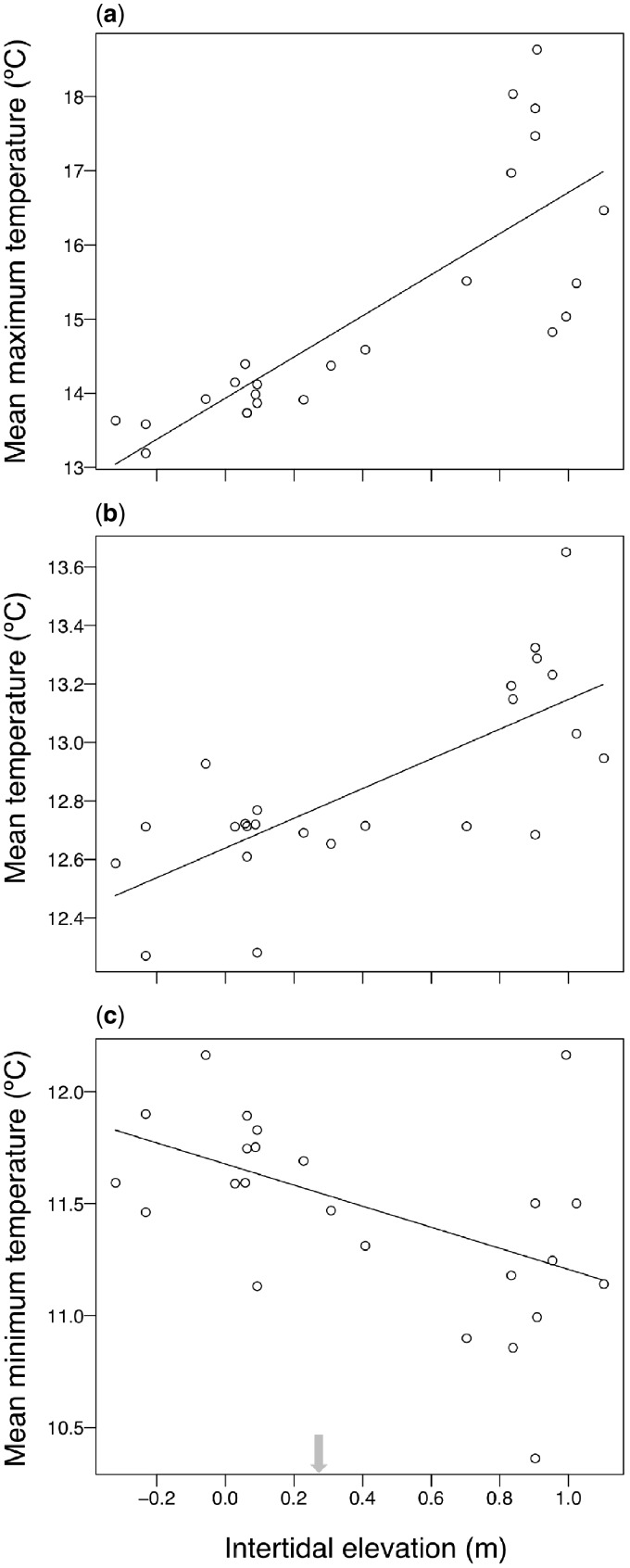
Mean under-rock temperature over the entire sampling period. Each point represents one rock. (**a**) Mean maximum temperature. (**b**) Mean temperature. (**c**) Mean minimum temperature. Note difference in *y* axis scale. The intertidal elevation boundary between the MIZ and HIZ is indicated with an arrow.

The HIZ has much greater spatial thermal variation than the MIZ, particularly with respect to daily thermal maxima. The SD of mean daily maximum temperature among rocks in the HIZ (4.1°C) was more than twice that in the MIZ (1.8°C; see also Fig. 2). Daily differences between the warmest and coolest maxima among rocks in the HIZ averaged 5.2°C and sometimes reached over 25°C. In contrast, daily differences in the warmest and coolest maxima among MIZ rocks averaged only 1.8°C, and never exceeded 19°C.

**Fig. 3:**
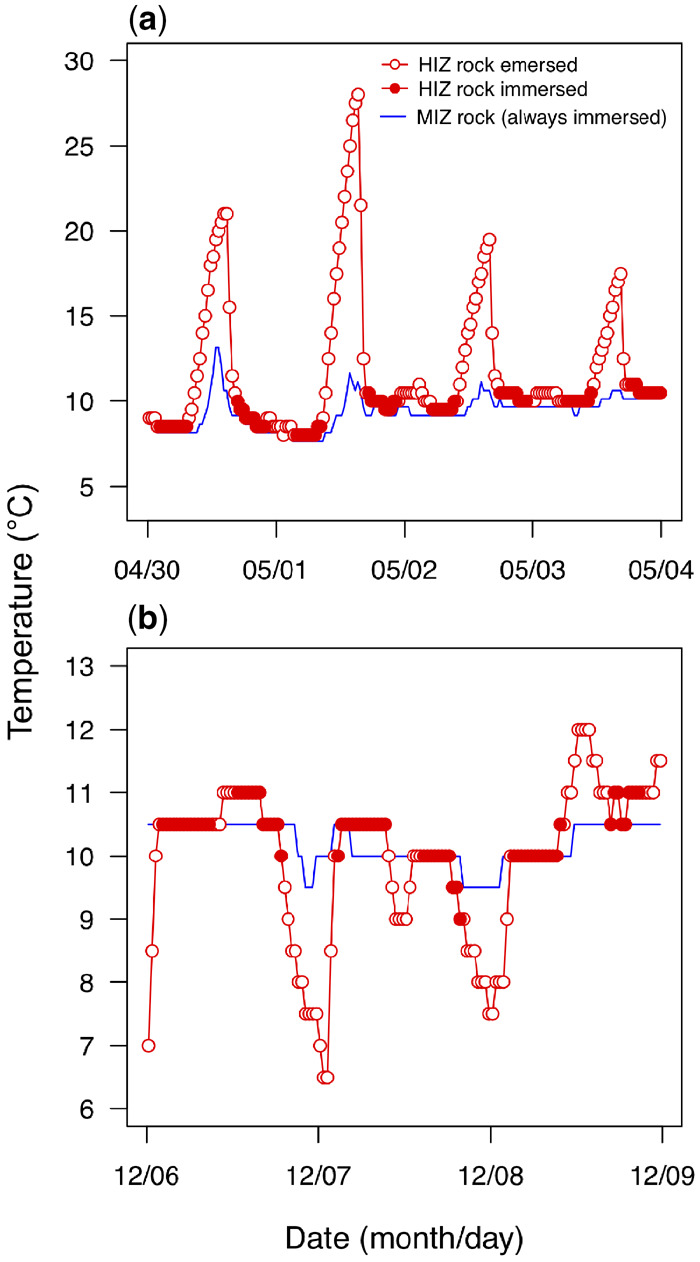
Under-rock temperatures for an HIZ and MIZ rock over several days in (**a**) May 2016 and (**b**) December 2016. Records are separated by whether the HIZ rock was predicted to be immersed or emersed. The MIZ rock was predicted to be immersed for all times shown.

Combining data across zones, rock intertidal elevation significantly influenced under-rock temperatures. Mean maximum (*P* < 0.001; *R*^2^ = 0.65; Fig. 2a) and overall mean (*P* < 0.001; *R*^2^ = 0.51; Fig. 2b) were positively associated with intertidal elevation, while the association was negative for mean minimum temperature (*P* = 0.012; *R*^2^ = 0.27; Fig. 2c). The importance of emersion time in determining thermal extremes can be demonstrated with several days of concurrent temperature data for two rocks: one regularly emersed rock in the HIZ (elevation 1.0 m) and one rarely emersed rock in the MIZ (−0.2 m elevation; Fig. 3). Daytime emersion of the HIZ rock in spring led to a steady increase in temperature before a steep decline with immersion (Fig. 3a). In contrast, nighttime emersion of the HIZ rock during the winter, when air temperature is low, led to steady decreases in temperature before a sharp increase at immersion (Fig. 3b). In both cases, the immersed MIZ rock temperature fluctuates much less. Neither rock area nor volume was associated with mean, mean daily maximum, or mean daily minimum rock temperatures in either zone (all *P* > 0.05), with one exception: rocks of larger volume in the MIZ had lower mean daily maxima (*P* = 0.005; Supplementary Fig. S5).

### Metabolic expenditure

**Fig. 4:**
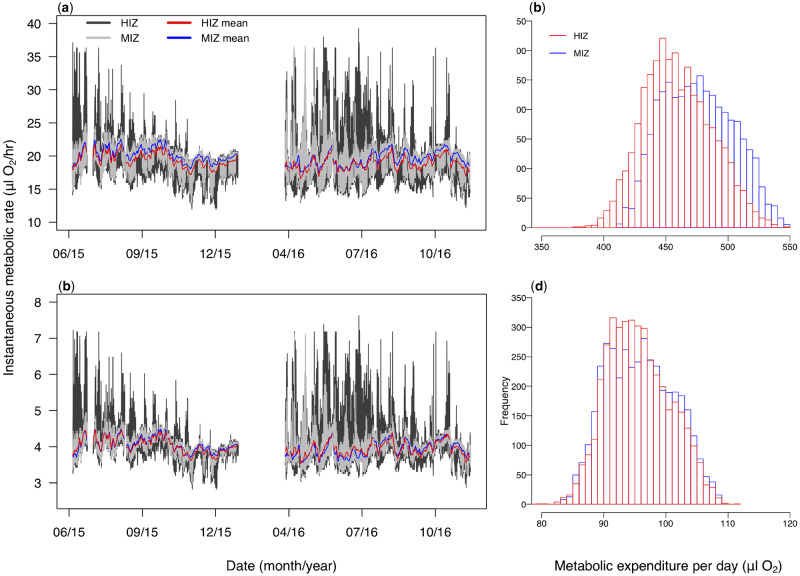
Predicted aerobic metabolism for the crab *P. cinctipes* based on under-rock temperatures measured in the HIZ and MIZ. Predicted instantaneous routine metabolic rates for (**a**) a large crab (4.3 g) and (**b**) a small crab (0.9 g) (Stillman and Somero 1996). Histograms of predicted cumulative daily routine energy expenditure for (**c**) a large and (**d**) a small crab.

Mean instantaneous metabolic rates were similar between zones for both large (4.3 g; MIZ mean±SD =19.9 ± 1.5 µL O_2_/h; HIZ =19.2 ± 2.3 µL O_2_/h; Fig. 4a) and small crabs (0.9 g; MIZ = 4.0 ± 0.3 µL O_2_/h; HIZ = 4.0 ± 0.4 µL O_2_/h; Fig. 4b). Mean daily maximum instantaneous metabolic rates were also similar between zones, though slightly higher in the HIZ (large crabs, HIZ = 22.1 ± 1.0 µL O_2_/h, MIZ = 20.9 ± 0.2 µL O_2_/h; small crabs, HIZ = 4.50 ± 0.22 µL O_2_/h, MIZ = 4.18 ± 0.05 µL O_2_/h). Mean cumulative daily metabolic expenditures were also similar between zones, though values skewed higher in the MIZ for large crabs (MIZ = 475.9 ± 28.4 µL O_2_; HIZ = 459.3 ± 27.5 µL O_2_; Fig. 4c), but not small crabs (MIZ = 95.8 ± 5.4 µL O_2_; HIZ = 95.5 ± 5.1 µL O_2_; Fig. 4d).

Among-rock variation in metabolic traits only differed substantially between zones for maximum instantaneous metabolism, as the SD was approximately four times greater among HIZ rocks than MIZ rocks for both large (1.0 vs. 0.2 µL O_2_/h) and small crabs (0.22 vs. 0.05 µL O_2_/h). Differences among rocks in the HIZ were particularly pronounced on the warmest days. For example, on some days a large crab under the warmest HIZ rock would experience an instantaneous metabolic rate over 30 µL O_2_/h higher than it would under the coolest HIZ rock, while in the MIZ differences among rocks were never greater than 20 µL O_2_/h.

### Heat tolerance and temperature-dependent behavior

**Fig. 5:**
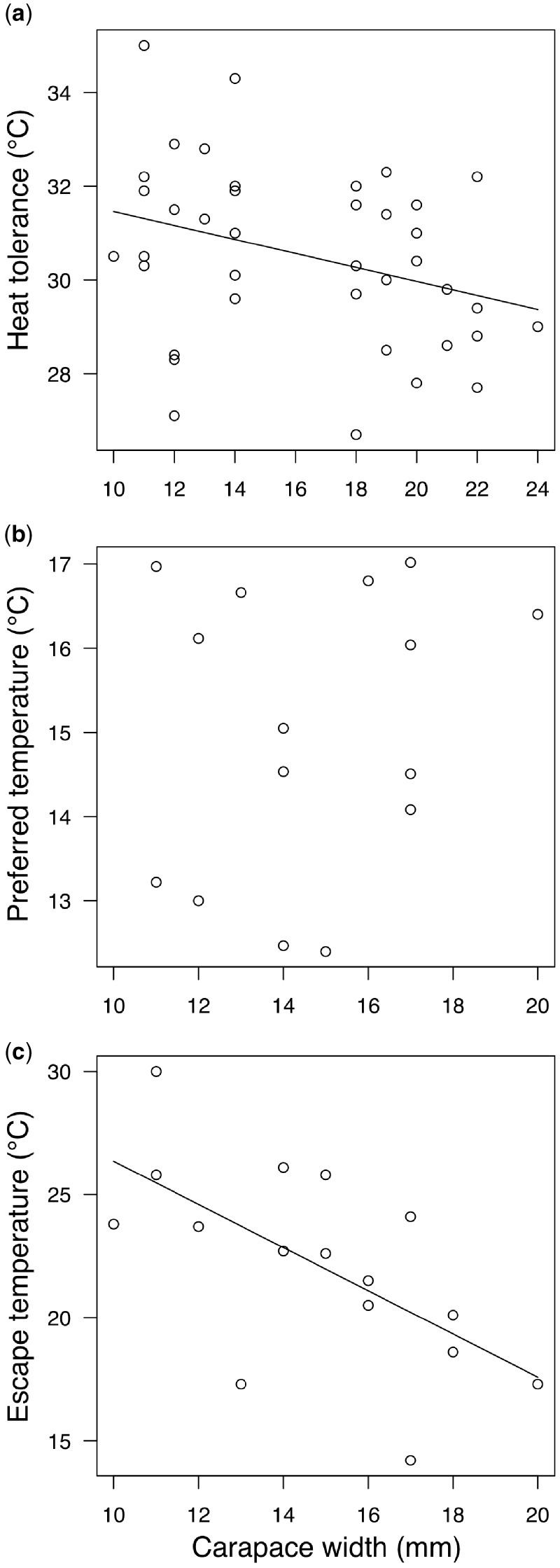
Size-dependent physiology and behavior of *P. cinctipes*. (**a**) Cardiac heat tolerance. (**b**) Preferred temperature. (**c**) Escape temperature (i.e., the temperature at which a crab moves to avoid heat).

The heat tolerance of crabs was (mean ± standard error [SE]) 30.5°C ± 0.3°C. However, heat tolerance was size-dependent. Large crabs were less heat tolerant than small crabs (Heat tolerance [°C] = 33.0–0.14 × carapace width; *R*^2^ = 0.11, *P* = 0.041; Fig. 5a).

The thermal preference of *P. cinctipes* was 15.0°C ± 0.4°C and unrelated to body size (*R*^2^ = 0.05, *P* = 0.431; Fig. 5b). The escape temperature of crabs was 20.5°C ± 0.6°C, though as with heat tolerance, it was size dependent: large crabs moved at lower temperatures than small crabs (*T*_esc_ = 35.12–0.88 × carapace width; *R*^2^ = 0.39, *P* = 0.009; Fig. 5c).

### Demography

**Fig. 6:**
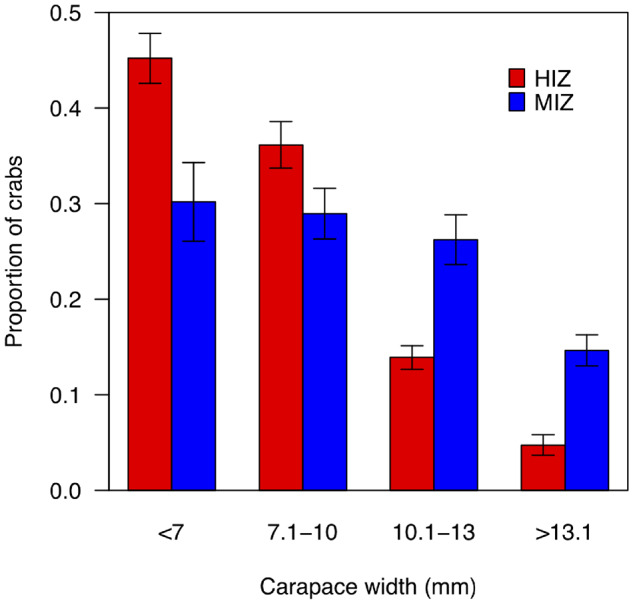
Summary of *P. cinctipes* demographic size structure by intertidal zone. Bars represent SE.

Total crab density was higher within the MIZ (mean±SE: 108.6 ± 10.9 crabs/m^2^) than the HIZ (43.4 ± 6.8 crabs/m^2^), as was the density of crabs of each size class (Supplementary Fig. S6). The MIZ consistently had proportionally more large crabs and fewer small crabs than the HIZ (Fig. 6).

### Simulated size-dependent demography

**Fig. 7:**
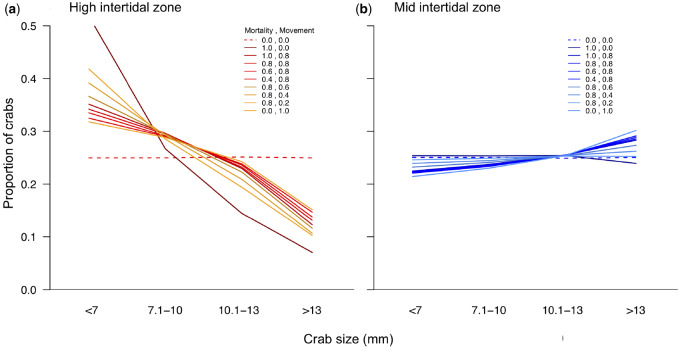
Individual-based simulations of demographic size structure in the (**a**) HIZ and (**b**) MIZ. Lines represent the mean result of 1000 simulations with a given parameter set for the probability that a crab dies (mortality) or moves (movement) if it experiences a temperature that reaches its predicted size-dependent heat tolerance or escapes temperature threshold, respectively. See “Methods” section for more details.

Simulations predicted proportionally more large crabs and fewer small crabs in the MIZ than in the HIZ (Fig. 7). The result was robust to different probabilities that crabs moved or perished upon experiencing size-dependent *T*_esc_ or CT_max_, and did not appear in null simulations with no size-dependence (Fig. 7; see Supplementary Table S2 for a summary of all simulation results).

## Discussion

How microclimates vary in space and time can affect many ecological and evolutionary processes (Gunderson and Leal 2012; Caillon et al. 2014; Hannah et al. 2014; Woods et al. 2015). We found clear patterns of spatial and temporal microclimatic variation between and within intertidal zones under rocks in boulder shore habitat, and demonstrate that this variation can have important physiological, behavioral, and demographic consequences for intertidal organisms. Furthermore, many of the consequences are body-size dependent. Below, we discuss these results and their broader implications.

### Thermal variability under intertidal rocks

Maximum daily temperatures were higher under HIZ than MIZ rocks, with the greatest differences during spring and summer low tides (Fig. 1b). However, overall mean temperatures in the HIZ and MIZ differed by only 0.3°C (Fig. 1a), likely for two reasons. First, both HIZ and MIZ rocks are immersed most of the time, when they share the same (water) temperature (Fig. 3a). Second, HIZ rocks reach slightly colder daily minima than MIZ rocks, particularly during the fall and winter months (Figs 1 and 3b). Colder minima in the HIZ probably result from longer exposure to cold night air and, on clear nights, radiative heat loss to the cold night sky (Fig. 3; Gates 1980). Thermal maxima and minima are associated with elevation and therefore emersion time, and the effect is greatest on maxima (Figs 2 and 3). This pattern predicts that organisms under HIZ rocks should have greater heat and cold tolerance than lower intertidal organisms, and there is evidence to support this prediction. For example, *P. cinctipes* is more heat and cold tolerant than *P. eriomerus*, a congener that lives in the low intertidal zone (Stillman and Somero 1996). Rock volume also affected thermal conditions under rocks, but only in the MIZ, and only with respect to maximum temperatures (Supplementary Fig. S5).

Spatial variation in heat extremes differed between zones, with a wider range of thermal conditions available among HIZ than MIZ rocks. Fine-scale spatial temperature variation has important implications for the expression of physiological traits (Jimenez et al. 2015) and dictates the potential for motile organisms to find suitable habitat and avoid stress through behavioral thermoregulation (Sears et al. 2016). Therefore, the patterns of thermal variability that we observed are likely to impact the rich diversity of taxa that occupy the spaces under rocks on boulder shores (e.g., Kuklinski and Barnes 2008; Chapman 2012; Liversage et al. 2017). We explore these potential consequences for *P. cinctipes* below.

### Metabolic consequences of under-rock thermal variability

Individuals in warmer habitats should have greater baseline aerobic metabolism, which can correspond to greater baseline energetic need and affect the energetic scope to invest in reproduction and other important functions (Pörtner and Knust 2007; Dillon et al. 2010; Miller et al. 2015). Accordingly, instantaneous metabolic rates were often much higher in the HIZ than in the MIZ (Fig. 4). Nonetheless, spatial thermal variation adds complexity to the baseline energetic consequences of occupying different zones, since crabs in the HIZ will experience very different acute metabolic demands depending on what rock they reside under, while this effect is dampened in the MIZ where there is less thermal variation. Interestingly, mean instantaneous metabolic rates were very similar between zones (Fig. 4a and b), and cumulative daily baseline metabolism was skewed toward greater energy consumption in the MIZ for large crabs (Fig. 4c). Three factors contribute to this counterintuitive result. First, high temperatures in the HIZ are short-lived. Second, HIZ rocks reach colder temperatures than MIZ rocks (Figs 1–3), which depresses metabolism. Third, *P. cinctipes* metabolism is depressed in air relative to water at a given temperature (Supplementary Fig. S4; Stillman and Somero 1996), and heat extremes occur during emersion.

Size-dependent differences in metabolism also complicate energetic predictions. In *P. cinctipes*, large crabs have greater metabolic depression in air than small crabs (Stillman and Somero 1996; Supplementary Fig. S4) and are therefore affected more by emersion. As noted above, daily baseline metabolic expenditure is skewed toward higher values in the MIZ for large, but not small, crabs (Fig. 4c and d). Reduced metabolism in air has been proposed to benefit organisms higher in the intertidal due to energetic savings (Marshall and McQuaid 1991; Sokolova and Pörtner 2001; Marshall et al. 2011; Marshall and McQuaid 2011). It is difficult to say in this case whether or not reduced metabolism is beneficial without more information. For example, if metabolic depression in air results from unavoidable constraints on oxygen acquisition (Pörtner 2012; Fusi et al. 2016), the capacity for physiological work toward important processes (e.g., assimilation and reproduction) would be reduced. Our findings reinforce the complexity and species specificity of assessing energetic costs in the intertidal zone, and of predicting how rising temperatures will impact energetic budgets.

### Interactions between body size and thermal variation

Overheating risk should be higher in the HIZ due to higher thermal maxima. Indeed, no rocks in the MIZ reached the mean overheating threshold of *P. cinctipes* (30.5°C), but rocks regularly did so in the HIZ, particularly during the spring and summer months (Fig. 1a). However, assessing overheating risk is complicated by the fact that small crabs are more heat tolerant than large crabs (Fig. 5a; see also Jensen and Armstrong 1991). In addition, large crabs were behaviorally less heat tolerant than small crabs (Fig. 5c). Size-dependent thermal inertia cannot explain these observations, because it would bias measurements toward greater heat tolerance in large animals due to their body temperatures lagging further behind environmental temperatures during a thermal ramp. Thermal inertia is unlikely to play a large role in our experiments in any case: porcelain crabs are small, disc shaped animals adapted to living in narrow air spaces under rocks, and therefore have large surface-area-to-volume ratios. Our results are consistent with growing evidence in marine organisms that small individuals are more heat tolerant than large individuals (Peck et al. 2009; Recsetar et al. 2012; Ohlberger 2013; Di Santo and Lobel 2017). We do not know why small crabs are more heat tolerant. It has been posited that large animals should be more prone to mismatches between oxygen supply and demand under warming due to the allometry of oxygen delivery systems (e.g., Forster et al. 2012; Cheung et al. 2013; Tirsgaard et al. 2015). However, there is still debate about the extent to which oxygen limitation sets heat limits (Ern et al., 2016). More work is clearly necessary to fully understand the mechanisms that underlie heat sensitivity of large individuals in aquatic habitats.

The size-dependent thermal responses of crabs predict that large animals should be underrepresented in the HIZ, where temperatures are more likely to reach heat tolerance and escape behavior thresholds. That is precisely what we found in our demographic data (Fig. 6). Furthermore, simulations parameterized by observed habitat temperatures and size-dependent trait values broadly recapitulated body size structure in the field (Fig. 7), particularly in the HIZ where thermal effects should predominate. The maintenance of some large individuals in the HIZ within our simulations is facilitated by the large spatial thermal variation in the HIZ: at the warmest times not all rocks within the HIZ reach physiological and behavioral thresholds, providing thermal refugia that can be behaviorally exploited (Huey et al. 2009; Hayford et al. 2015; Ng et al. 2017).

Spatial structure in body size across the intertidal zone is common within species (Robles 1987; Rochette et al. 2003; Pardo and Johnson 2005), and there are numerous potential explanations for these patterns (e.g., Vermeij 1972; Underwood 1984). Our results indicate that size-dependent thermal physiology could contribute to these patterns where large individuals are less common in the warmest microhabitats (Robles 1987; Takada 1996; Pardo and Johnson 2005). There are numerous potential implications of this finding. For example, interactions between size and temperature could help explain patterns of intra-specific competition in size-structured populations, with subsequent effects on spatial patterns of competitive interactions between species that influence population and community-level processes (Werner and Gilliam 1984; Ebenman and Persson 2012). These implications could extend to marine organisms that live outside of the intertidal zone, since increased heat sensitivity in large individuals is found in marine animals from many different habitat types (Daufresne et al. 2009; Peck et al. 2009; Recsetar et al. 2012; Ohlberger 2013; Clark et al. 2017; Di Santo and Lobel 2017; Lindmark et al. 2018).

## Conclusion

We found extensive microclimatic variation under intertidal boulders, and demonstrate that this variation can have significant ecological consequences for organisms that live under them (Huey et al. 1989). One of the major implications to emerge from our study is that microclimatic variation can interact with size-dependent phenotypic variation in ways that influence overheating risk, behavioral thermoregulation, and the demographic structure of populations (Denny et al. 2011). Size-dependent thermal physiology is well established in both terrestrial (e.g., Baudier et al. 2015; Klockmann et al. 2017) and aquatic organisms (see citations above). Therefore, it is important to consider this form of intraspecific variation when investigating the ecological consequences of microclimatic variation. Doing so can yield mechanistic insights that may be critical for predicting and mitigating the impacts of human activity as the globe continues to warm.

## Supplementary Material

obz024_Supplementary_Data
