# Supplementary material for: Hot Rocks and Not-So-Hot Rocks on the Seashore: Patterns and Body-Size Dependent Consequences of Microclimatic Variation in Intertidal Zone Boulder Habitat
Source: Integr Org Biol. 2019 Oct 9;1(1):obz024. doi: 10.1093/iob/obz024 (PMC7671146; doi:10.1093/iob/obz024)
Supplement: obz024_Supplementary_Data [file obz024_supplementary_data.pdf]

Supplementary Table 1. Demographic sampling effort in the high intertidal zone (HIZ) and mid-intertidal zone (MIZ) by date.

Date	# HIZ rocks sampled	# MIZ rocks sampled
7/6/15	3	3
8/2/15	12	5
8/17/15	11	-
8/30/15	12	5
9/13/15	11	-
10/12/15	11	-
10/26/15	11	4
11/9/15	9	-
1/9/16	10	-
1/22/16	15	15
2/20/16	20	-
4/10/16	8	11
5/8/16	9	13
5/26/16	9	13
6/8/16	9	13
7/7/16	9	10
7/22/16	8	13
8/3/16	9	13
10/17/16	9	3
12/13/16	9	13

Supplementary Table S2. Results of simulations to investigate the effects of habitat temperature and body size on the demographic characteristics of *Petrolisthes cinctipes* within different intertidal zones. Each proportion value is the average of 1000 simulations with a given probability that crabs perish or move ("Probability of overheating" and "Probability of moving," respectively) given that they reach their Critical thermal maximum or Escape temperature threshold based on our size-dependent empirical measurements.

		Proportion of crabs in each size category. Size category in mm							
		High intertidal zone				Mid intertidal zone			
Probability of overheating	Probability of moving	< 7.0	7.1 - 10.0	10.0-13.0	>13.1	< 7.0	7.1 - 10.0	10.0-13.0	>13.1
0	0	0.25	0.25	0.251	0.25	0.25	0.25	0.249	0.25
0	0.2	0.27	0.264	0.252	0.215	0.234	0.239	0.249	0.278
0	0.4	0.285	0.271	0.25	0.194	0.226	0.236	0.25	0.289
0	0.6	0.299	0.277	0.246	0.178	0.22	0.233	0.252	0.294
0	0.8	0.308	0.283	0.245	0.164	0.217	0.232	0.253	0.299
0	1	0.318	0.288	0.242	0.152	0.214	0.23	0.254	0.302
0.2	0	0.294	0.262	0.238	0.206	0.25	0.252	0.25	0.248
0.2	0.2	0.304	0.273	0.241	0.182	0.238	0.243	0.25	0.268
0.2	0.4	0.31	0.279	0.242	0.169	0.23	0.239	0.251	0.279
0.2	0.6	0.314	0.284	0.241	0.161	0.224	0.235	0.253	0.287
0.2	0.8	0.317	0.286	0.242	0.156	0.219	0.233	0.254	0.294
0.2	1	0.318	0.289	0.242	0.151	0.215	0.23	0.254	0.302
0.4	0	0.34	0.272	0.221	0.167	0.252	0.251	0.252	0.246
0.4	0.2	0.341	0.28	0.227	0.152	0.243	0.245	0.251	0.261
0.4	0.4	0.336	0.288	0.233	0.144	0.235	0.241	0.252	0.273
0.4	0.6	0.331	0.288	0.236	0.145	0.227	0.238	0.254	0.282
0.4	0.8	0.325	0.29	0.238	0.147	0.221	0.233	0.255	0.291
0.4	1	0.319	0.286	0.243	0.152	0.214	0.231	0.253	0.301
0.6	0	0.397	0.27	0.2	0.133	0.252	0.253	0.253	0.243
0.6	0.2	0.38	0.287	0.208	0.126	0.244	0.248	0.252	0.256
0.6	0.4	0.363	0.291	0.221	0.125	0.238	0.243	0.253	0.267
0.6	0.6	0.349	0.293	0.229	0.129	0.23	0.24	0.254	0.277
0.6	0.8	0.335	0.291	0.236	0.138	0.222	0.236	0.254	0.288
0.6	1	0.317	0.288	0.243	0.152	0.215	0.23	0.253	0.302
0.8	0	0.453	0.275	0.174	0.099	0.253	0.253	0.253	0.241
0.8	0.2	0.418	0.285	0.194	0.103	0.246	0.249	0.253	0.252
0.8	0.4	0.392	0.293	0.209	0.107	0.239	0.244	0.254	0.262
0.8	0.6	0.366	0.295	0.223	0.116	0.232	0.241	0.254	0.273
0.8	0.8	0.342	0.295	0.232	0.131	0.223	0.236	0.255	0.285
0.8	1	0.318	0.287	0.244	0.151	0.215	0.231	0.253	0.302
1	0	0.518	0.267	0.145	0.07	0.254	0.253	0.254	0.239
1	0.2	0.459	0.282	0.176	0.083	0.249	0.25	0.253	0.248
1	0.4	0.42	0.293	0.196	0.091	0.241	0.246	0.254	0.259
1	0.6	0.384	0.299	0.214	0.103	0.234	0.242	0.255	0.269
1	0.8	0.351	0.296	0.23	0.123	0.225	0.237	0.255	0.283
1	1	0.318	0.288	0.243	0.151	0.214	0.23	0.254	0.302

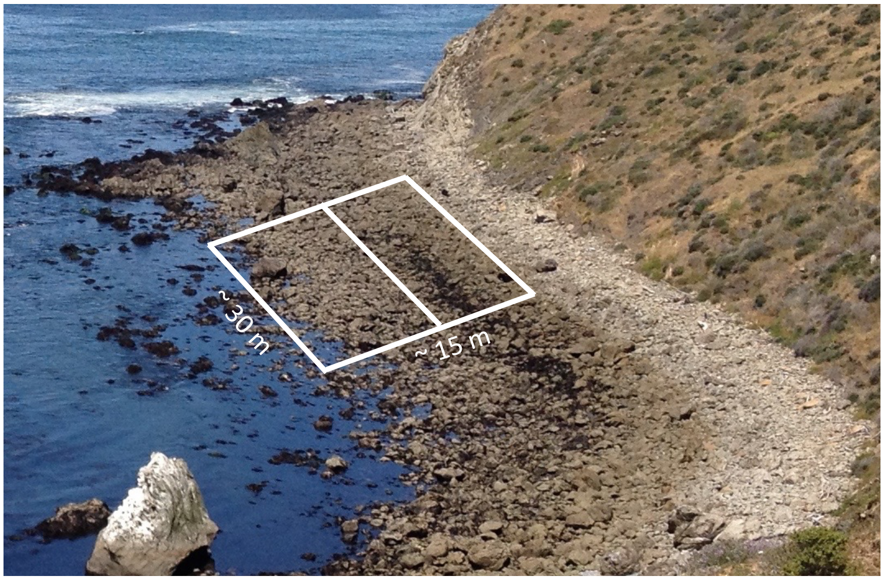

Supplementary figure S1. The shoreline on which temperature and crab demographic data were collected during an afternoon low tide event, Sonoma County, CA (38°30′45.79′′N, 123°14′45.58′′W). The outline of the study area is shown in white lines and separated into our intertidal zone designations, with the mid intertidal zone area closest to the ocean. Photo by A.R. Gunderson.

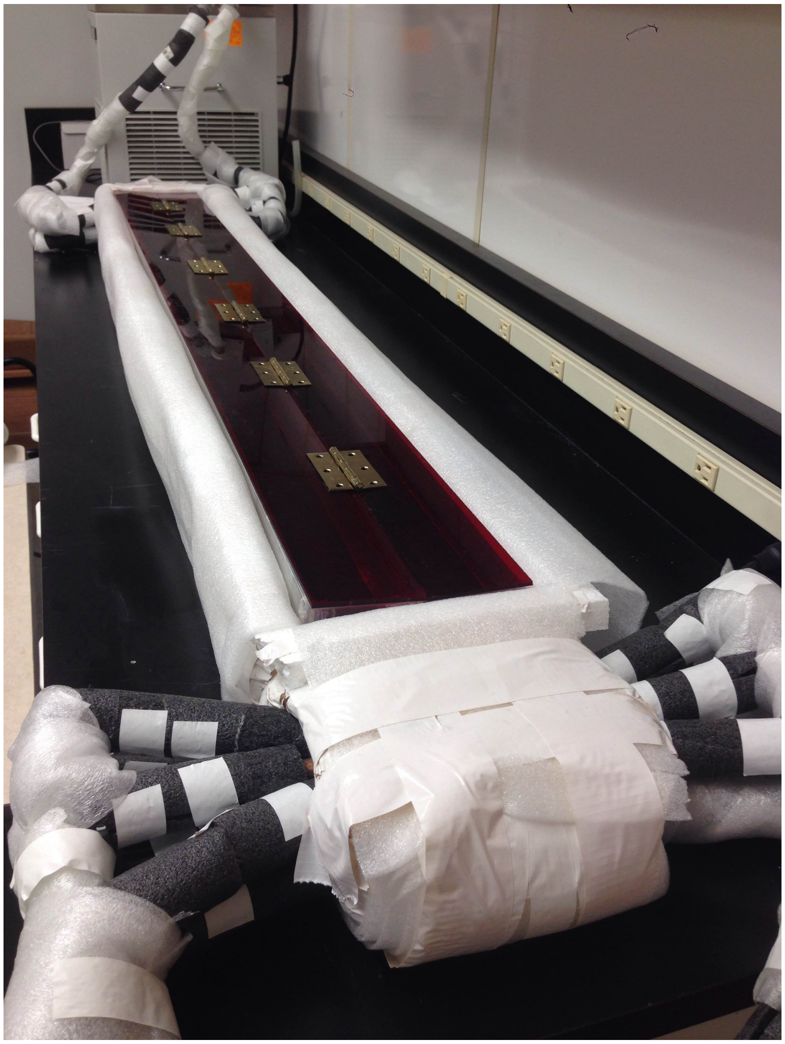

Supplementary Figure S2. Thermal gradient bar used to measure the preferred temperature of *Petrolisthes cinctipes*.

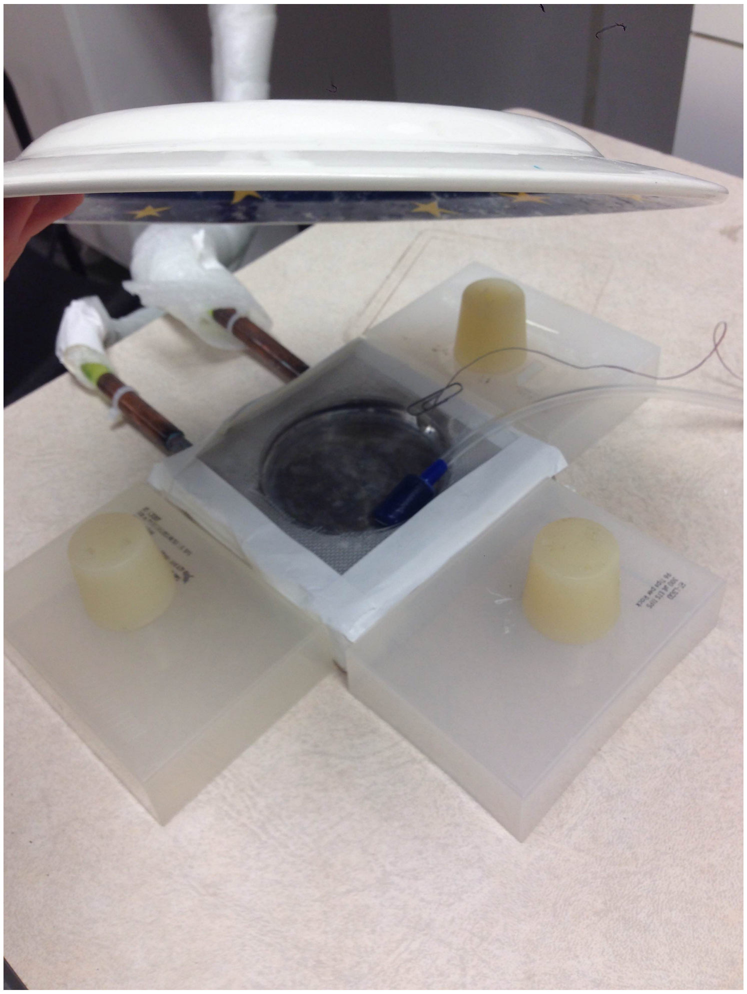

Supplementary Figure S3. Apparatus used to measure escape temperature of *Petrolisthes cinctipes*.

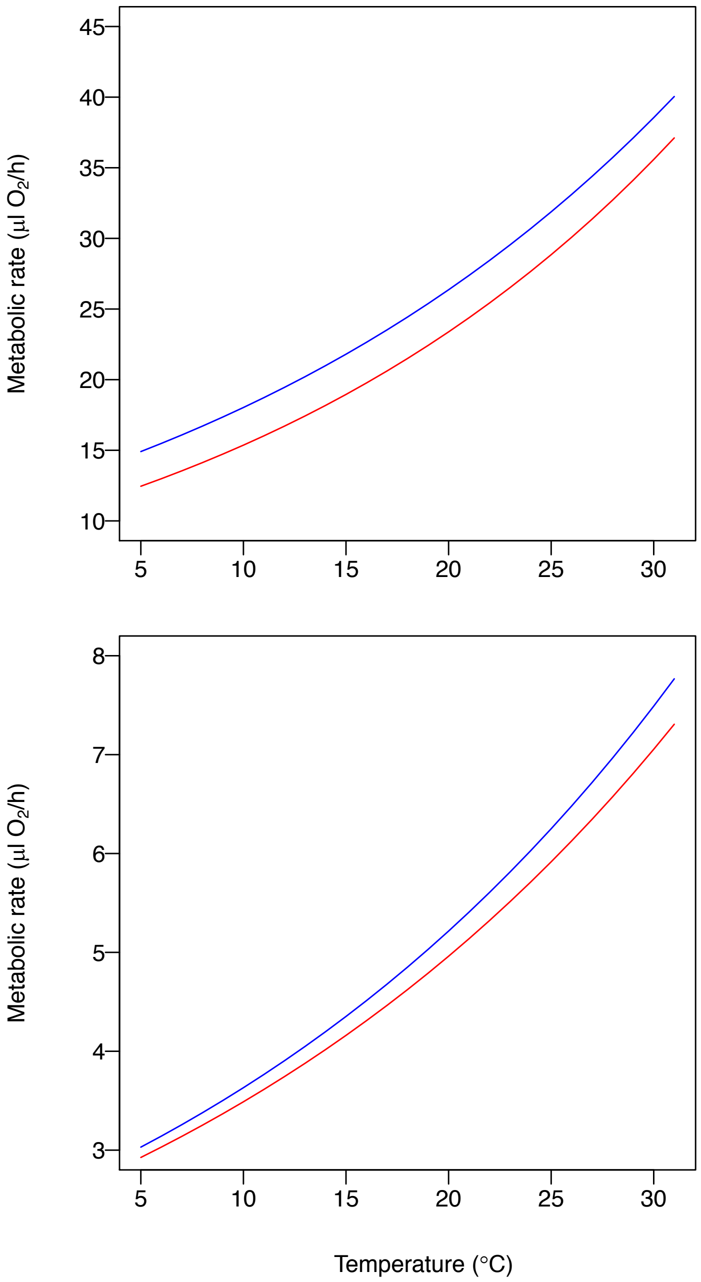

Supplementary figure S4. Routine metabolic rates in water (blue) and air (red). Top, large crab (4.3 g). (Bottom, small crab (0.9 g). Curves were fit to data from Stillman and Somero (1996).

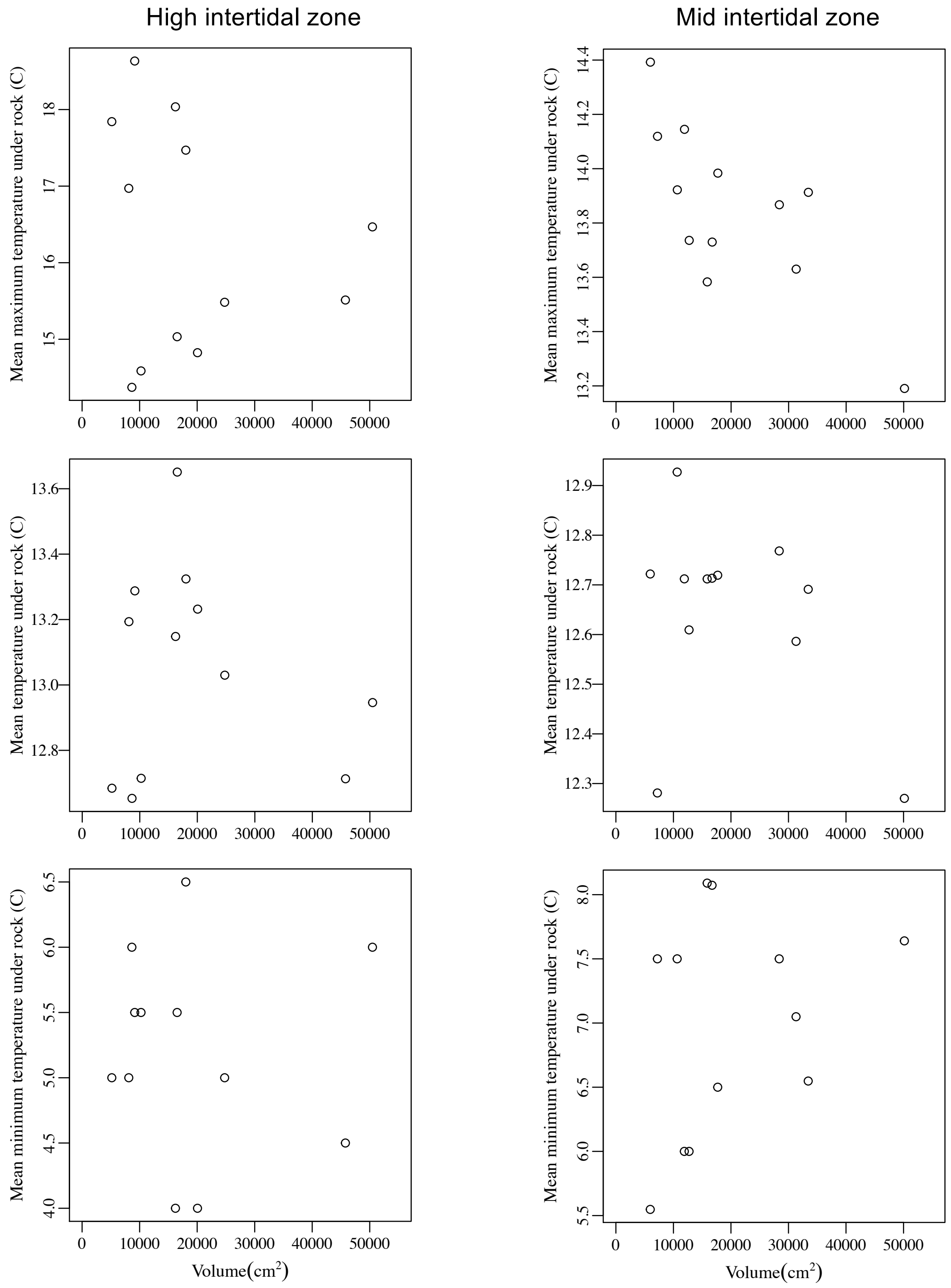

Supplementary Figure S5. Relationship between rock volume and under-rock temperatures in the high intertidal zone and mid intertidal zone. Rock volume was only associated with mean maximum rock temperature in the Mid intertidal zone (top right).

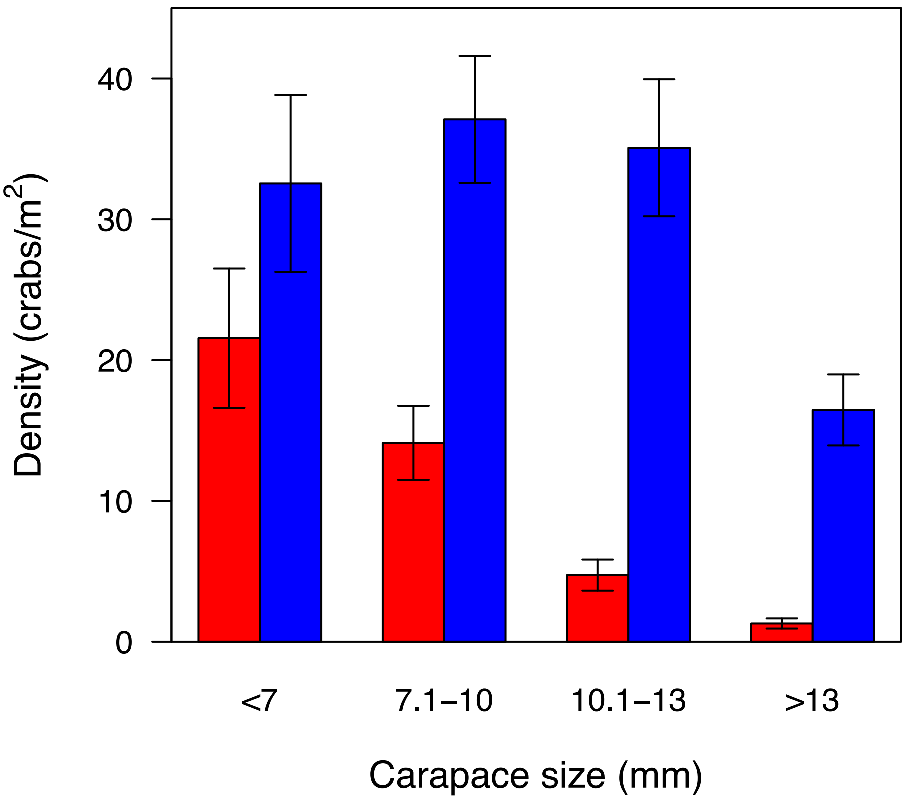

Supplementary Figure S6. Density of crabs of different size categories under rocks in the high intertidal zone (red) and mid intertidal zone (blue).

Supplementary appendix 1. R-code used to predict tide height at Fort Ross State Park, CA, at every time point for which we have a temperature record. Packages used are *rtides* and *lubridate*.

```
alltht<-NULL ## where all of the tide heights will be stored
for(i in levels(alldat$rock)){
  tide<-NULL
  tide<-subset(alldat[alldat$rock == i,], select = c(cdat)) # getting the dates with temp records
  tide$Station<-rep(tide_stations()[21], nrow(tide)) ## add the station
  names(tide)<-c("DateTime", "Station") # naming that function requires
  tide$DateTime<-with_tz(as.POSIXct(tide$DateTime), "PST8PDT")
  tide$DateTime<-tide$DateTime+hours(7)+minutes(13) ## adding time so output is in the same timezone as our site, minus the 13 minute difference between Point Arena and Fort Ross. Functions from lubridate
  tht<-tide_height_data(tide, harmonics = rtide::harmonics) # getting the tide heights.
  alltht<-c(alltht, tht$TideHeight) # add current rock tide heights to others
}
```
